# Pulmonary artery pressure monitoring in chronic heart failure: effects across clinically relevant subgroups in the MONITOR-HF trial

**DOI:** 10.1093/eurheartj/ehae323

**Published:** 2024-05-11

**Authors:** Pascal R D Clephas, Victor W Zwartkruis, Jishnu Malgie, Marco W F van Gent, Hans-Peter Brunner-La Rocca, Mariusz K Szymanski, Vokko P van Halm, M Louis Handoko, Wouter E M Kok, Folkert W Asselbergs, Roland R J van Kimmenade, Olivier C Manintveld, Nicolas M D A van Mieghem, Saskia L M A Beeres, Marco C Post, C Jan Willem Borleffs, Raymond Tukkie, Arend Mosterd, Gerard C M Linssen, Ruud F Spee, Mireille E Emans, Tom D J Smilde, Jan van Ramshorst, Charles J H J Kirchhof, Margriet W Feenema-Aardema, Carlos A da Fonseca, Mieke van den Heuvel, Ronald Hazeleger, Martijn van Eck, Loek van Heerebeek, Eric Boersma, Michiel Rienstra, Rudolf A de Boer, Jasper J Brugts

**Affiliations:** Department of Cardiology, Thorax Centre, Cardiovascular Institute, Erasmus University Medical Center, Dr. Molewaterplein 40, 3015GD Rotterdam, Netherlands; Department of Cardiology, University Medical Centre Groningen, Groningen, Netherlands; Department of Cardiology, Thorax Centre, Cardiovascular Institute, Erasmus University Medical Center, Dr. Molewaterplein 40, 3015GD Rotterdam, Netherlands; Department of Cardiology, Albert Schweitzer Hospital, Dordrecht, Netherlands; Department of Cardiology, Maastricht University Medical Centre, Maastricht, Netherlands; Department of Cardiology, University Medical Center Utrecht, Utrecht, Netherlands; Department of Cardiology, Amsterdam Cardiovascular Sciences, Amsterdam University Medical Centre, University of Amsterdam, Amsterdam, Netherlands; Department of Cardiology, University Medical Center Utrecht, Utrecht, Netherlands; Department of Cardiology, Amsterdam Cardiovascular Sciences, Amsterdam University Medical Centre, University of Amsterdam, Amsterdam, Netherlands; Department of Cardiology, Amsterdam Cardiovascular Sciences, Amsterdam University Medical Centre, University of Amsterdam, Amsterdam, Netherlands; Department of Cardiology, Radboud University Medical Centre, Nijmegen, Netherlands; Department of Cardiology, Thorax Centre, Cardiovascular Institute, Erasmus University Medical Center, Dr. Molewaterplein 40, 3015GD Rotterdam, Netherlands; Department of Cardiology, Thorax Centre, Cardiovascular Institute, Erasmus University Medical Center, Dr. Molewaterplein 40, 3015GD Rotterdam, Netherlands; Department of Cardiology, Leiden University Medical Centre, Leiden, Netherlands; Department of Cardiology, University Medical Center Utrecht, Utrecht, Netherlands; Department of Cardiology, St. Antonius Hospital, Nieuwegein, Netherlands; Department of Cardiology, HAGA Hospital, Den Haag, Netherlands; Department of Cardiology, Spaarne Hospital, Haarlem, Netherlands; Department of Cardiology, Meander Medical Centre, Amersfoort, Netherlands; Department of Cardiology, Hospital Group Twente, Almelo, Netherlands; Department of Cardiology, Maxima Medical Centre, Veldhoven/Eindhoven, Netherlands; Department of Cardiology, Ikazia Hospital, Rotterdam, Netherlands; Department of Cardiology, Scheeper Hospital TREANT, Emmen, Netherlands; Department of Cardiology, Noordwest Hospital Group, Alkmaar, Netherlands; Department of Cardiology, Alrijne Hospital, Leiderdorp, Netherlands; Department of Cardiology, Medical Centre Leeuwarden, Leeuwarden, Netherlands; Department of Cardiology, Medical Centre Leeuwarden, Leeuwarden, Netherlands; Department of Cardiology, Medisch Spectrum Twente, Enschede, Netherlands; Department of Cardiology, Vie Curi Hospital, Venlo, Netherlands; Department of Cardiology, Jeroen Bosch Hospital, ‘s-Hertogenbosch, Netherlands; Department of Cardiology, OLVG Hospital, Amsterdam, Netherlands; Department of Cardiology, Thorax Centre, Cardiovascular Institute, Erasmus University Medical Center, Dr. Molewaterplein 40, 3015GD Rotterdam, Netherlands; Department of Cardiology, University Medical Centre Groningen, Groningen, Netherlands; Department of Cardiology, Thorax Centre, Cardiovascular Institute, Erasmus University Medical Center, Dr. Molewaterplein 40, 3015GD Rotterdam, Netherlands; Department of Cardiology, Thorax Centre, Cardiovascular Institute, Erasmus University Medical Center, Dr. Molewaterplein 40, 3015GD Rotterdam, Netherlands

**Keywords:** Randomized controlled trial, Pulmonary artery pressure, Sensor, Telemonitoring, Subgroups, Quality of life

## Abstract

**Background and Aims:**

In patients with chronic heart failure (HF), the MONITOR-HF trial demonstrated the efficacy of pulmonary artery (PA)-guided HF therapy over standard of care in improving quality of life and reducing HF hospitalizations and mean PA pressure. This study aimed to evaluate the consistency of these benefits in relation to clinically relevant subgroups.

**Methods:**

The effect of PA-guided HF therapy was evaluated in the MONITOR-HF trial among predefined subgroups based on age, sex, atrial fibrillation, diabetes mellitus, left ventricular ejection fraction, HF aetiology, cardiac resynchronization therapy, and implantable cardioverter defibrillator. Outcome measures were based upon significance in the main trial and included quality of life-, clinical-, and PA pressure endpoints, and were assessed for each subgroup. Differential effects in relation to the subgroups were assessed with interaction terms. Both unadjusted and multiple testing adjusted interaction terms were presented.

**Results:**

The effects of PA monitoring on quality of life, clinical events, and PA pressure were consistent in the predefined subgroups, without any clinically relevant heterogeneity within or across all endpoint categories (all adjusted interaction *P*-values were non-significant). In the unadjusted analysis of the primary endpoint quality-of-life change, weak trends towards a less pronounced effect in older patients (*P*_interaction_ = .03; adjusted *P*_interaction_ = .33) and diabetics (*P*_interaction_ = .01; adjusted *P*_interaction_ = .06) were observed. However, these interaction effects did not persist after adjusting for multiple testing.

**Conclusions:**

This subgroup analysis confirmed the consistent benefits of PA-guided HF therapy observed in the MONITOR-HF trial across clinically relevant subgroups, highlighting its efficacy in improving quality of life, clinical, and PA pressure endpoints in chronic HF patients.

## Structured Graphical Abstract

**Structured Graphical Abstract:**
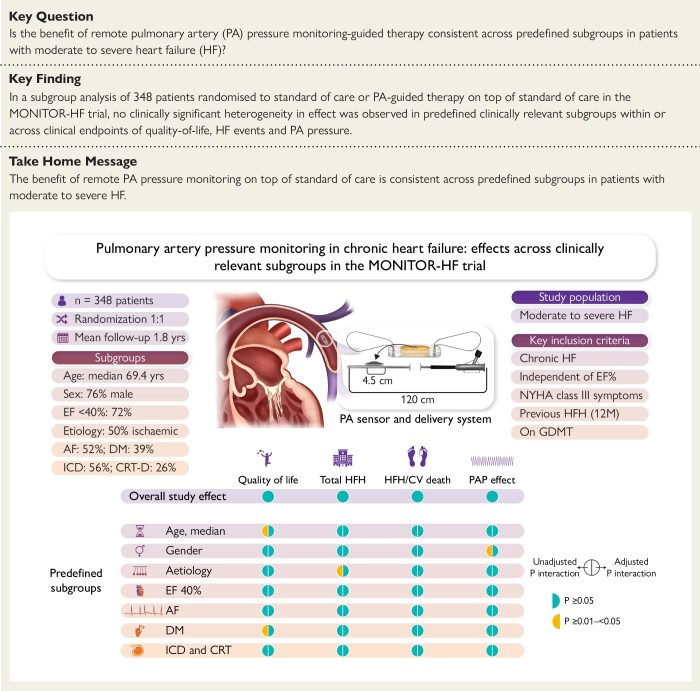
The overview figure presents the *P*-values associated with the interaction terms on each endpoint per subgroup. Green corresponds to an overall significant effect in the main trial, and for the predefined subgroups to no statistically significant interaction term on the associated clinical endpoint. The HFH/CV death endpoint refers to a time to event analysis of the composite endpoint HFH or CV death. AF, atrial fibrillation; CV, cardiovascular; DM, diabetes mellitus; EF, ejection fraction; GDMT, guideline-directed medical therapy; ICD, implantable cardiac defibrillator; CRT, cardiac resynchronization therapy; HF, heart failure; HFH, heart failure hospitalization; PA, pulmonary artery; PAP, pulmonary artery pressure.

**See the editorial comment for this article ‘Pulmonary artery pressure-guided heart failure care: the setting matters’, by S. Störk, https://doi.org/10.1093/eurheartj/ehae441.**

## Introduction

In response to the frequent hospitalizations caused by heart failure (HF),^1,2^ remote haemodynamic monitoring has emerged as one of the potential strategies to reduce HF hospitalizations (HFH) and alleviate the burden HF poses on healthcare systems.^3^ Pulmonary artery (PA) pressure has shown to be a clinically meaningful haemodynamic parameter linked to intracardiac filling pressure,^4^ with PA-guided HF therapy demonstrating consistent results in reducing HFH.^5^ The CHAMPION and GUIDE-HF trials, along with several post-approval studies, reported significant reductions in HFH with the CardioMEMS HF system.^6–12^ The MONITOR-HF trial has reported a significant improvement in quality of life and similar reductions in HFH with this device.^13^ Aggregated data from these randomized trials, as reported in a recent meta-analysis that combined these three CardioMEMS HF system trials, underscore the benefits of PA-guided HF therapy.^14^

While these results are promising, it is important to determine which patients benefit most from PA-guided HF therapy, as the success of this remote HF management tool may be influenced by relevant patient characteristics. Some of these patient characteristics may have haemodynamic interactions with HF, while other patient characteristics may interfere with the mode of the effect of remote haemodynamic monitoring, which relies on timely pharmacological interventions based on fluctuations in PA pressure over time.

Prior subgroup analyses have already been performed with data from the CHAMPION trial,^15,16^ the post-approval study,^17–19^ and the MEMS-HF study,^20^ showing consistent effects of PA-guided HF therapy. However, the effect of PA-guided therapy on quality of life as a primary outcome has not yet been assessed among subgroups. Additionally, some of these previous subgroup analyses are scattered over several separate analyses, were sometimes performed on single or selected HF outcomes, and did not always include interaction terms to assess differential effects.

In this comprehensive pre-specified subgroup analysis, we therefore aimed to investigate the consistency of the PA-guided HF therapy effects observed in MONITOR-HF across relevant predefined HF subgroups based on age, sex, atrial fibrillation (AF), diabetes mellitus (DM), left ventricular ejection fraction (LVEF), HF aetiology, cardiac resynchronization therapy (CRT) device, and implantable cardiac defibrillator (ICD). Additionally, a selected number of non-predefined subgroup analyses, based on previous studies and clinical relevancy, were included as exploratory analyses.^17,19,20^

## Methods

### Trial design and participants

The MONITOR-HF trial was an open-label, randomized controlled trial (RCT) conducted between 2019 and 2023 in 25 participating hospitals in the Netherlands, of which the main results have previously been published.^13^ Briefly, MONITOR-HF enrolled patients with New York Heart Association (NYHA) Class III chronic HF and a previous HFH or emergency visit with the necessity of intravenous diuretics in the previous 12 months. Patients with HF with a reduced ejection fraction (HFrEF) were eligible for enrolment when treated with optimal or maximally tolerated guideline-directed medical therapy (GDMT) and evaluated for an ICD or CRT. Participants were 1:1 randomized to either standard HF management (standard of care group) or standard HF management with the addition of PA-guided HF therapy using the CardioMEMS HF system (PA-guided HF therapy group). All analyses are based on the intention-to-treat population. The full inclusion and exclusion criteria are included in Supplementary data online, *Table S1*.

### Definition of subgroups

The following subgroups were predefined in MONITOR-HF:^13^ age (≥69.4 vs. < 69.4 years), sex (female vs. male), HF aetiology (ischaemic vs. non-ischaemic), baseline LVEF (≥40% vs. < 40%), AF (yes vs. no), DM (yes vs. no), CRT (yes vs. no), and ICD (yes vs. no). The subgroups for age were based on the median age observed in MONITOR-HF. Left ventricular ejection fraction values were obtained from echocardiography measurements at baseline. Atrial fibrillation was defined as a documented history of AF (paroxysmal or permanent).

In addition, the following subgroups were included as exploratory analyses: baseline mean PAP (mPAP) during implant (>25 vs. ≤ 25 mmHg), baseline renal function (estimate glomerular filtration rate [eGFR] ≥ 60 vs. < 60 mL/min), baseline obesity (body mass index [BMI] > 30 vs. ≤30 kg/m^2^), GDMT used at baseline in HFrEF patients (≥3 vs. < 3 GDMT drug classes) and baseline N-terminal pro-B-type natriuretic peptide (NT-proBNP) (≥2146 pg/mL vs. < 2146 pg/mL). Complementary to the baseline mean PA pressure subgroup, the PA pressure endpoints were also assessed for baseline pulmonary vascular resistance (PVR) during implant (>2 WU vs. ≤ 2 WU). The baseline mean PA pressure and PVR values were obtained from Swan–Ganz measurements that were done during implantation of the CardioMEMS HF system and were only available in the PA-guided HF therapy group. The baseline mean PA pressure subgroups were based on the definition of pulmonary hypertension (PH) of the 2015 ESC/ERS Guidelines for the diagnosis and treatment of PH (mean PA pressure >25 mmHg) and both the instructions from the CardioMEMS HF system and the MONITOR-HF treatment guideline where action was needed when the mean PA pressure exceeded 25 mmHg.^13,21^ Renal function subgroups were based on the threshold for chronic kidney disease (CKD) in MONITOR-HF (eGFR < 60 mL/min),^13^ and eGFR was based on creatinine clearance. Obesity was defined as a BMI > 30 kg/m^2^ in line with the World Health Organization definition.^22^ The NT-proBNP subgroups were based on the median NT-proBNP observed in MONITOR-HF at baseline.

### Endpoints

MONITOR-HF reported significant effects of PA-guided HF therapy for Kansas City Cardiomyopathy Questionnaire (KCCQ) overall summary score difference at 12 months and difference in change between baseline and 12 months.^13^ Although there is some overlap between the two quality of life endpoints, both offer relevant insights. The absolute difference in KCCQ overall summary score at 12 months provides insight into the overall quality of life after 12 months of follow-up at group level, while the difference in mean change KCCQ overall summary score analyses the quality of life by considering the difference between baseline and 12 months of follow-up, which is conditional on individuals having a score on both time points in a paired analysis. MONITOR-HF also reported significant effects of PA-guided HF therapy on total HFH, composite of total HFH and all-cause mortality, time to first HFH, and composite of time to first HFH or cardiovascular (CV) mortality. The HFH endpoints include both HFH and urgent visits (according to trial definitions). Only 8.5% of events were urgent visits (11 in the PA-guided therapy group and 17 in the standard-of-care group). Regarding PA pressure endpoints, we studied the mean PA pressure area under the curve (AUC), and mean PA pressure changes up to 12 months post-implant. This subgroup analysis assessed the consistency of the overall effect on the endpoints that were significant in the main trial among the previously described subgroups. While procedural complications were low in MONITOR-HF (2.3% device and/or system-related complications), we assessed them for any differences among subgroups.

### Statistical analysis

Within-group changes between baseline and 12 months in mean KCCQ overall summary scores were analysed with a paired *t*-test. Between-group differences in KCCQ overall summary score at 12 months and KCCQ overall summary score mean change were analysed with an unpaired *t*-test.^13^ The Andersen–Gill extension of the Cox regression model with the robust sandwich estimate of variance was used for the total HFH and composite of total HFH and all-cause mortality analyses, and a regular Cox proportional hazards regression model was used for the time to first HFH and composite of time to first HFH or CV mortality analyses. The mean PA pressure AUC was analysed by calculating the AUC of the time and magnitude the PA pressure was above or below the baseline PA pressure, defined as the average of the first seven readings at home,^6,13^ using the trapezoidal rule.^6,13^ Lastly, the change between baseline and 12 months in mean PA pressure was analysed with a paired *t*-test. For the baseline mean PA pressure and PVR subgroups, in contrast with the other subgroups, only the within-group results were shown.

Interaction effects were calculated with interaction terms within regression models (allocated treatment group x subgroup characteristic) for KCCQ overall summary score difference at 12 months and difference in change, total HFH, composite of total HFH and all-cause mortality, time to first HFH, and composite of time to first HFH or CV mortality. The mean PA pressure AUC, and mean PA pressure change analyses were only performed in the PA-guided HF therapy group making interaction term calculations in regression models impossible. The interaction effects for these analyses were therefore defined as between subgroup differences and were calculated with an unpaired *t*-test.

*P*-values for interaction were adjusted to account for the inflated Type I error (false positives) due to multiple testing (multiple subgroups and multiple endpoints).^23^*P*-values were adjusted for the number of tests within an endpoint (both pre- and not pre-specified) with the Holm procedure,^24^ a similar but more powerful version of the established Bonferroni procedure.^25^ Both unadjusted and adjusted *P*-values for interaction were calculated and presented.

When considering adjustment for multiple testing in multiple subgroup comparisons, it is important to acknowledge the diversity in approaches. Some advocate for adjusting for the number of comparisons or adopting a higher significance threshold, emphasizing the importance of considering the number of comparisons when drawing statistical inferences, especially in the case of multiple endpoints.^26^ Conversely, others argue that utilizing interaction tests already addresses significant concerns regarding multiple comparisons,^27^ and that adjusting for multiple testing may further reduce statistical power.^28^ We have chosen to present both unadjusted and adjusted *P*-values for interaction. Although adjusted *P*-values offer a more conservative estimate due to inflated Type I error, unadjusted *P*-values may serve as hypothesis-generating if clinically relevant or with a plausible clinical mechanism. Therefore, in view of any clinical relevance of findings, we also studied multiple endpoints to verify consistency of effects across multiple clinical pathways and endpoint categories (quality of life-, clinical-, and PA pressure endpoints).

## Results

The baseline characteristics of the MONITOR-HF study are provided in Supplementary data online, *Table S2*, and the baseline medical therapy use of the HFrEF patients is included in Supplementary data online, *Table S3*. Detailed results of all endpoints are provided in Supplementary data online, *Tables S4–S6*.

### Kansas City Cardiomyopathy Questionnaire overall summary score endpoints

**Figure 1:**
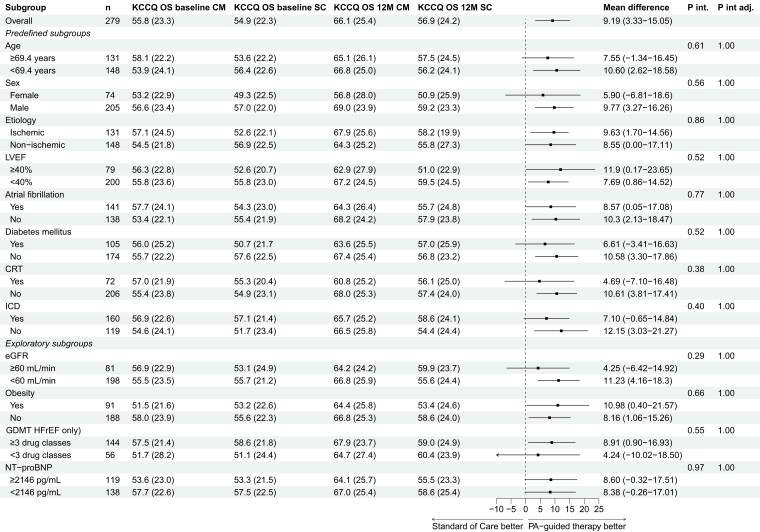
Kansas City Cardiomyopathy Questionnaire overall summary score difference at 12 months. KCCQ OS, Kansas City Cardiomyopathy Questionnaire overall summary score; CM, CardioMEMS (PA-guided therapy) group; SC, standard of care group; P int, P interaction; P int adj, P interaction adjusted; 12M, 12 months; LVEF, left ventricular ejection fraction; CRT, cardiac resynchronization therapy; ICD, implantable cardiac defibrillator; eGFR, estimated glomerular filtration rate; GDMT, guideline-directed medical therapy; HFrEF, heart failure with a reduced ejection fraction

**Figure 2:**
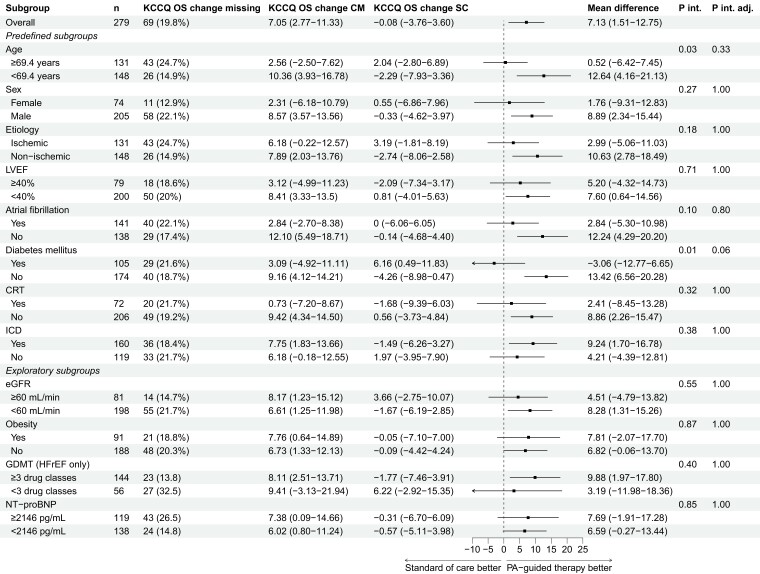
Kansas City Cardiomyopathy Questionnaire overall summary score difference in change between baseline and 12 months. KCCQ OS, Kansas City Cardiomyopathy Questionnaire overall summary score; CM, CardioMEMS (PA-guided therapy) group; SC, standard of care group; P int, P interaction; P int adj, P interaction adjusted; LVEF, left ventricular ejection fraction; CRT, cardiac resynchronization therapy; ICD, implantable cardiac defibrillator; eGFR, estimated glomerular filtration rate; GDMT, guideline-directed medical therapy; HFrEF, heart failure with a reduced ejection fraction

The results of the KCCQ overall summary score difference at 12-months endpoint are provided in *Figure 1*. There was consistency across all subgroups in favour of PA-guided HF therapy, without any signs of interaction effects. *Figure 2* presents the results of the endpoint KCCQ overall summary score difference in change between baseline and 12 months. While most subgroups demonstrated no significant interaction on the effect of PA-guided HF therapy, only patients with and without DM showed a borderline non-significant interaction after adjusting (*P*_interaction_ = 0.01; adjusted *P*_interaction_ = 0.06). Additionally, older patients showed a less pronounced effect as compared to younger patients, with a difference in KCCQ overall summary score change of 0.52 (95% confidence interval [CI] −6.42–7.45) vs. 12.64 (95% CI 4.16–21.13), however, the interaction was not significant after adjusting for multiple testing (*P*_interaction_ = .03; adjusted *P*_interaction_ = .33). Also, the number of missing KCCQ overall summary score change values in older patients was substantially higher than in younger patients (24.7% vs. 14.9%).

### Clinical endpoints

**Figure 3:**
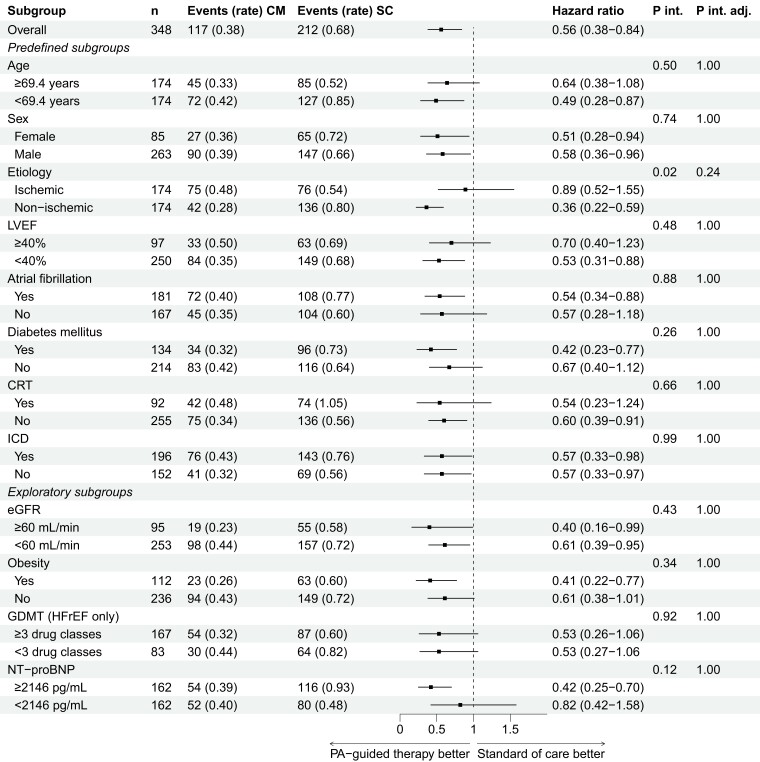
Total heart failure hospitalizations. Rate, events/patient-year; CM, CardioMEMS (PA-guided therapy) group. SC, standard of care group; P int, P interaction; P int adj, P interaction adjusted; LVEF, left ventricular ejection fraction; CRT, cardiac resynchronization therapy; ICD, implantable cardiac defibrillator; eGFR, estimated glomerular filtration rate; GDMT, guideline-directed medical therapy; HFrEF, heart failure with a reduced ejection fraction. Adapted from Brugts *et al.*^13^ with permission from Elsevier

**Figure 4:**
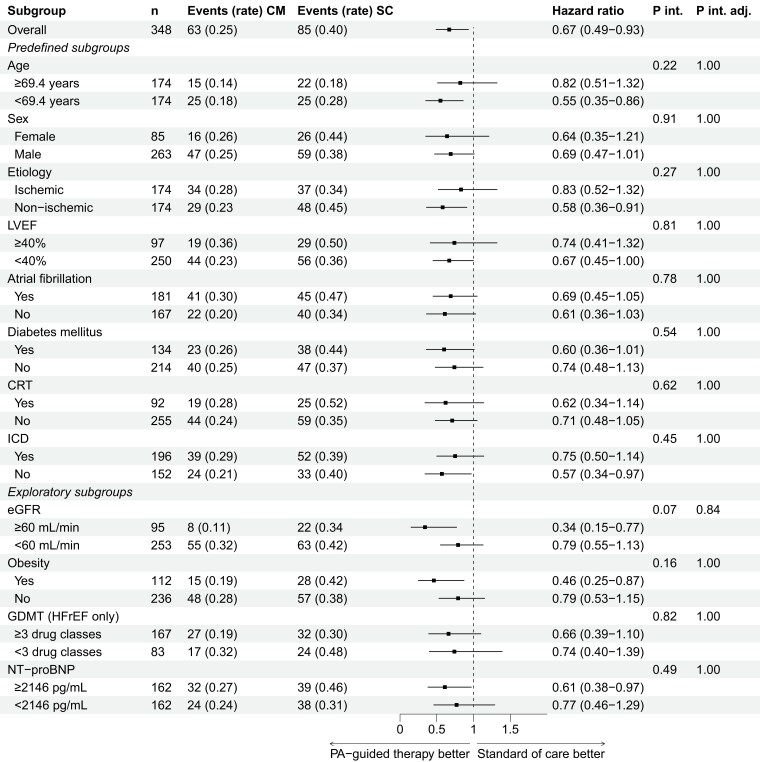
Time to first heart failure hospitalization. Rate, events/patient-year; CM, CardioMEMS (PA-guided therapy) group. SC, standard of care group; P int, P interaction; P int adj, P interaction adjusted; LVEF, left ventricular ejection fraction; CRT, cardiac resynchronization therapy; ICD, implantable cardiac defibrillator; eGFR, estimated glomerular filtration rate; GDMT, guideline-directed medical therapy; HFrEF, heart failure with a reduced ejection

The results of the endpoint total HFH are depicted in *Figure 3*. All subgroups exhibited a consistent reduction in HFH, aligning with the overall outcome. Notably, in the unadjusted analysis, an interaction effect for aetiology was observed that did not persist in the adjusted analysis (*P*_interaction_ = .02; adjusted *P*_interaction_ = .24); patients with a non-ischaemic and ischaemic aetiology had a hazard ratio of 0.36 (95% CI 0.22–0.59) and 0.89 (95% CI 0.52–1.55), respectively. In *Figure 4*, the results for the time to first HFH are presented. In line with the total HFH endpoint, all subgroups showed a consistent effect of PA-guided HF therapy without any observed interaction effects. The results of the composite endpoint of total HFH and all-cause mortality are included in Supplementary data online, *Figure S1*. Lastly, the results of the composite endpoint of time to first HFH or CV mortality are included in Supplementary data online, *Figure S2*.

Analysing these endpoints without urgent visits yielded similar results. Lastly, there were no significant differences in procedural complications among the subgroups.

### Pulmonary artery pressure endpoints

**Figure 5:**
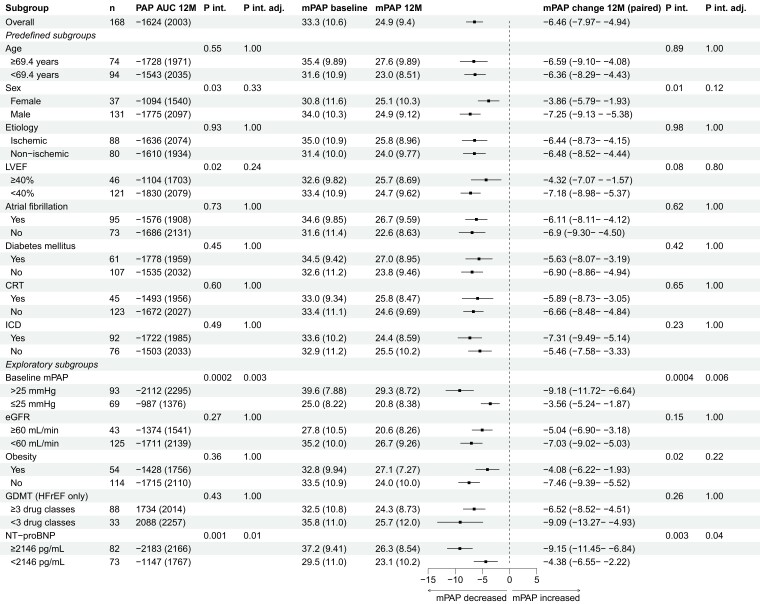
Mean pulmonary artery pressure area under the curve and mean pulmonary artery pressure change between baseline and 12 months. PAP AUC, pulmonary artery pressure area under the curve (mmHg.days); mPAP, mean pulmonary artery pressure (mmHg); P int, P interaction; P int adj, P interaction adjusted; 12M, 12 months; LVEF, left ventricular ejection fraction; AF, atrial fibrillation; DM, diabetes mellitus; CRT, cardiac resynchronization therapy; ICD, implantable cardiac defibrillator; eGFR, estimated glomerular filtration rate; GDMT, guideline-directed medical therapy; HFrEF, heart failure with a reduced ejection

The differences in mean PA pressure AUC and mean PA pressure change between baseline and 12 months are presented in *Figure 5*. Across all subgroups, a decrease in the mean PA pressure AUC was observed, consistent with the overall effect. An interaction was observed in the unadjusted analysis for LVEF, that did not persist in the adjusted analysis (*P*_interaction_ = .02; adjusted *P*_interaction_ = .24); patients with an LVEF ≥ 40% and <40% had an AUC of −1104 (1703) mmHg.day and −1830 (2079) mmHg.day, respectively.

Consistent with the overall reductions in mean PA pressure AUC, all subgroups exhibited a reduction in mean PA pressure change. For sex, an interaction was observed in the unadjusted analysis that also did not persist in the adjusted analysis (*P*_interaction_ = .01; adjusted *P*_interaction_ = .12); female and male patients had a change in mean PA pressure of −3.86 (95% CI −5.79–−1.93) and −7.25 (95% CI −9.13–−5.38) mmHg, respectively.

### Exploratory subgroup analyses

The exploratory subgroup analyses (eGFR, obesity, GDMT use in HFrEF, baseline NT-proBNP, and baseline mean PA pressure) did not show any clinically relevant interaction effects in the quality of life, clinical and PA pressure endpoints. However, a trend was observed for obesity in the unadjusted analysis of the mean PA pressure change endpoint that disappeared in the adjusted analysis (*P*_interaction_ = .02; adjusted *P*_interaction_ = .22); patients with and without obesity had a mean PA pressure change of −4.08 (95% CI −6.22–−1.93) and −7.46 (95% CI −9.39–−5.52), respectively. Logically, the change in mean PA pressure was higher in patients with a baseline mean PA pressure >25 mm Hg with a change of −9.18 (95% CI −11.72–−6.64) mmHg as compared to patients with a baseline mean PA pressure at baseline ≤25 mmHg with a change of −3.56 (95% CI −5.24–−1.87) mmHg. The same was observed for patients with an NT-proBNP ≥ 2146 pg/mL with a change of −9.15 (95% CI −11.45–−6.84) mmHg vs. patients with an NT-proBNP < 2146 pg/mL with a change of −4.38 (95% CI −6.55–−2.22) mmHg. For patients with a PVR > 2 and ≤2, this change was −8.49 (95% CI −10.94 – −6.05) mmHg and −5.85 (−8.03 – −3.68) mmHg, respectively. Further details on the exploratory subgroups are included in Supplementary data online, *Tables S4–S6*.

Patients with a baseline mean PA pressure > 25 and ≤ 25 mmHg had a mean KCCQ overall summary score at 12 months of 63.8 (SD 25.0) and 66.8 (SD 25.9), respectively. Moreover, both baseline mean PA pressure subgroups had improvements in the KCCQ overall summary score, with improvements of 6.89 (95% CI 1.09–12.70) and 7.46 (95% CI 0.56–14.36) for patient with a baseline mean PA pressure of >25 and ≤25 mmHg, respectively.

For the total HFH endpoint, patients with a baseline mean PA pressure >25 and ≤25 mmHg experienced 67 events (0.43/patient-year) and 46 events (0.34/patient-year) during follow-up, respectively. Both subgroups had a lower event rate than the overall standard of care group with 212 events (0.68/patient-year), indicating a consistent effect. Similar results were observed for the other clinical endpoints.

## Discussion

In this pre-specified subgroup analysis, we have demonstrated a consistent benefit of PA-guided HF therapy across a wide variety of relevant HF subgroups, in line with the results observed in the MONITOR-HF trial (*Structured Graphical Abstract*). No clinically relevant interaction effects were observed within or across clinical endpoint categories. This is the first pre-specified analysis of PA-guided HF therapy RCT data that provides a complete overview of all relevant endpoints and subgroups, while also including novel data on quality of life (KCCQ) endpoints.

As expected, patients with a baseline mean PA pressure >25 mmHg exhibited both a higher reduction in AUC and mean PA pressure change, also after adjusting for multiple testing. Interestingly, patients with a baseline mean PA pressure ≤25 mmHg still had reductions in AUC and mean PA pressure. Both baseline mean PA subgroups had a comparable improvement in quality of life and event rates of clinical endpoints that were lower than the overall standard of care group.

Potential identification of patients or subgroups who are most likely to benefit from PA-guided HF therapy is useful, as patient selection becomes more important with invasive telemonitoring techniques in comparison to non-invasive telemonitoring techniques.^3,29^ Additionally, in the ongoing transition phase within healthcare, where there is a shift from conventional healthcare models to encompassing remote care services,^30^ it is helpful to know which patients and subgroups can really benefit from remote care.^4^ Importantly, in this analysis the interaction terms did not identify certain patient groups that are more or less likely to benefit from PA-guided therapy. The findings of this analysis therefore instil confidence in the benefit of PA-guided HF therapy in the studied patients, given the consistent benefits observed across all clinically relevant HF subgroups. Several cost-effectiveness analyses also confirmed the cost-effective benefit of PA-guided therapy,^31–34^ and a recent meta-analysis of implantable haemodynamic monitors showed a significant improvement in long-term survival.^35^ The transition to remote care can be accelerated even more by effective integration in current healthcare workflows and reduction in physical outpatient visits of stable remote monitored patients.^31^

CHAMPION and GUIDE-HF also performed several subgroup analyses, of which the results were generally in line with this analysis. In GUIDE-HF, a significant interaction was found for NYHA class and sex for the composite endpoint of HFH and all-cause mortality, with NYHA Class II–III and female patients having a lower risk as compared with NYHA Class IV and male patients.^6^ However, these interaction effects were not observed in CHAMPION as stated by the authors of GUIDE-HF. In addition, both GUIDE-HF and CHAMPION found consistent effects of PA-guided HF therapy across all LVEF subgroups in separate subgroup analyses, without any observed interaction effects in the clinical and PA pressure endpoints.^15,16,36^

Finally, no significant interactions were found for NYHA class and sex, or any other subgroups, in a meta-analysis of CHAMPION, GUIDE-HF, and MONITOR-HF.^14^

A subgroup analysis from the German MEMS-HF study found comparable effects of PA-guided HF therapy regarding quality of life and clinical endpoints in patients with and without PH, and, in line with this analysis, a lower mean PA pressure AUC and a higher mean PA pressure reduction in patients with PH.^20^ The post-approval study (US) in turn found that PA-guided HF therapy reduced HFH, mean PA pressure AUC, and mean PA pressure in male and female patients and in patients with and without CKD or obesity.^17–19^ Interaction effects were only assessed in the obesity subgroup analysis, where none were found.^17^

Although we did not find significant interaction effects for the primary outcome on quality of life after adjusting for multiple testing, two crude trends appeared in the unadjusted analysis with weak associations. Older patients and patients with diabetes might experience less improvement in quality of life, likely due to the relation between frailty and increased comorbidities.^37–40^ However, these statistical interactions did not persist after adjusting for multiple testing, possibly influenced by higher missing data among older patients, partly due to mortality, or a chance finding. Nonetheless, older patients and patients with diabetes still benefited similarly from PA-guided HF therapy across the clinical HFH outcomes and PA pressure effects.

An overview of the analysis across the three types of endpoints—quality of life, HFH, and mean PA reduction—no subgroup displayed any clinically relevant heterogeneity or emerged as suitable for clinical selection for patients most likely to benefit from remote haemodynamic monitoring. Notably, we observed a reduction in mean PA pressures across all subgroups, complementing the observations as an intermediate of clinical effects. While some weak statistical trends were observed in the unadjusted analysis on quality of life for some subgroups with a higher comorbidity burden (elderly diabetics), this would be a call for particular engagement in the management of these comorbidities, and rather not be an argument to withhold pulmonary artery pressure (PAP) monitoring as the clinical effects on outcome and mean PAP were sustained and consistent.

### Strengths and limitations

This analysis has several strengths as compared to previous analyses in a number of key aspects. First, by consolidating all relevant HF subgroups into a single comprehensive analysis, this study avoids the fragmentation seen in previous research, providing a more unified and inclusive understanding of the efficacy of PA-guided HF therapy. Second, unlike some previous analyses that only reported on a selection of relevant HF outcomes, this study encompasses all endpoints where PA-guided HF therapy demonstrated benefit in the main analysis, ensuring a thorough intention-to-treat analysis of its clinical effectiveness on significant endpoints of the main trial. Third, in accordance with established recommendations regarding subgroup analyses of RCTs,^41^ the subgroups assessed were pre-defined, with any additional exploratory analyses that were added because of clinical relevancy clearly labelled and reported separately. Fourth, the inclusion of interaction terms to assess subgroup effects, along with the calculation and presentation of both adjusted and unadjusted *P*-values, further enhances the robustness and reliability of the findings.^41^ Finally, this analysis presents novel data by also including quality-of-life endpoints.

This analysis has several limitations. First, MONITOR-HF was an open-label RCT with a relatively small sample size of 348 patients which limits statistical power. Consequently, only a limited number (comprising the most important) of subgroups were evaluated, as smaller, more specific subgroups would have been too underrepresented for meaningful analysis. Second, the primary endpoint on mean KCCQ overall summary score change was assessed in a paired analysis and not adjusted for the baseline value in the randomized treatment arms. Third, the unadjusted analyses may have had an inflated Type I error (false positives) due to the numerous statistical tests performed considering the number of subgroups and endpoints that were assessed. However, we mitigated this by adjusting the analyses for multiple testing within each endpoint, although this may increase the risk of a Type II error (false negatives).^28^ We report both unadjusted and adjusted *P*-values for interaction and additionally evaluated—in light of clinical relevance—heterogeneity across the three endpoint categories. Finally, while three subgroups were not pre-defined, we included them as exploratory analyses due to their clinical relevance and previous subgroup analyses. We also maintained a clear distinction between pre-defined and exploratory subgroups throughout the analysis.

## Conclusions

In conclusion, this pre-specified subgroup analysis of the MONITOR-HF trial confirms the consistent effects of PA-guided HF therapy. Notably, improvements in quality of life-, clinical-, and PA pressure endpoints were observed consistently across most subgroups, indicating the robustness of PA-guided HF therapy in managing chronic HF. These findings underscore the overall efficacy and clinical applicability of PA-guided HF therapy in improving outcomes for patients with symptomatic chronic HF, providing valuable insights for the implementation of remote haemodynamic monitoring.

## Supplementary data

Supplementary data are available at *European Heart Journal* online.

## Supplementary Material

ehae323_Supplementary_Data

## Declarations

### Data Availability

No aggregate or patient-level collected data in this study can be made available externally owing to internal regulations, patient consent, and data regulations for outside Erasmus Medical Center. Yet, researchers interested in collaboration should contact the corresponding author.

### Pre-registered Clinical Trial Number

This trial was prospectively registered under the clinical trial registration number NTR7673 (NL7430) on the International Clinical Trials Registry Platform.
