# Supplementary material for: Pulmonary artery pressure monitoring in chronic heart failure: effects across clinically relevant subgroups in the MONITOR-HF trial
Source: Eur Heart J. 2024 May 11;45(32):2954–64. doi: 10.1093/eurheartj/ehae323 (PMC11335373; doi:10.1093/eurheartj/ehae323)
Supplement: ehae323_Supplementary_Data [file ehae323_supplementary_data.pdf]

Supplementary material

Figure S1. Total heart failure hospitalizations and all-cause mortality.

Subgroup	n	Events (rate) CM	Events (rate) SC	Hazard ratio	P int.	P int. adj.
Overall	348	159 (0.52)	257 (0.82)	0.63 (0.44−0.90)		
Predefined subgroups						
Age					0.60	1.00
≥69.4 years	174	66 (0.48)	116 (0.71)	0.70 (0.45−1.09)		
<69.4 years	174	93 (0.55)	141 (0.94)	0.58 (0.34−0.97)		
Sex					0.67	1.00
Female	85	33 (0.44)	73 (0.80)	0.56 (0.30−1.02)		
Male	263	126 (0.54)	184 (0.83)	0.65 (0.43−1.00)		
Etiology					0.02	0.24
Ischemic	174	104 (0.66)	102 (0.72)	0.93 (0.58−1.47)		
Non−ischemic	174	55 (0.37)	155 (0.91)	0.41 (0.25−0.67)		
LVEF					0.30	1.00
≥40%	97	44 (0.67)	71 (0.78)	0.84 (0.50−1.41)		
<40%	250	114 (0.47)	186 (0.84)	0.57 (0.37−0.90)		
Atrial fibrillation					0.76	1.00
Yes	181	95 (0.53)	129 (0.92)	0.60 (0.39−0.93)		
No	167	64 (0.50)	128 (0.74)	0.67 (0.37−1.23)		
Diabetes mellitus					0.67	1.00
Yes	134	57 (0.53)	118 (0.90)	0.58 (0.35−0.97)		
No	214	102 (0.51)	139 (0.77)	0.68 (0.43−1.09)		
CRT					0.33	1.00
Yes	92	52 (0.60)	93 (1.32)	0.52 (0.25−1.10)		
No	255	107 (0.49)	162 (0.67)	0.72 (0.49−1.04)		
ICD					0.70	1.00
Yes	196	97 (0.54)	171 (0.90)	0.61 (0.38−0.99)		
No	152	62 (0.48)	86 (0.70)	0.69 (0.43−1.11)		
Exploratory subgroups						
eGFR					0.28	1.00
≥60 mL/min	95	23 (0.28)	65 (0.69)	0.41 (0.17−0.97)		
<60 mL/min	253	136 (0.61)	192 (0.88)	0.69 (0.47−1.02)		
Obesity					0.92	1.00
Yes	112	37 (0.42)	70 (0.67)	0.61 (0.35−1.05)		
No	236	122 (0.56)	187 (0.90)	0.63 (0.41−0.98)		
GDMT (HFrEF only)					0.93	1.00
≥3 drug classes	167	72 (0.42)	101 (0.70)	0.61 (0.33−1.12)		
<3 drug classes	83	42 (0.61)	85 (1.09)	0.56 (0.30−1.04)		
NT−proBNP					0.16	1.00
≥2146 pg/mL	162	82 (0.59)	143 (1.14)	0.51 (0.32−0.82)		
<2146 pg/mL	162	66 (0.50)	94 (0.57)	0.89 (0.49−1.59)		

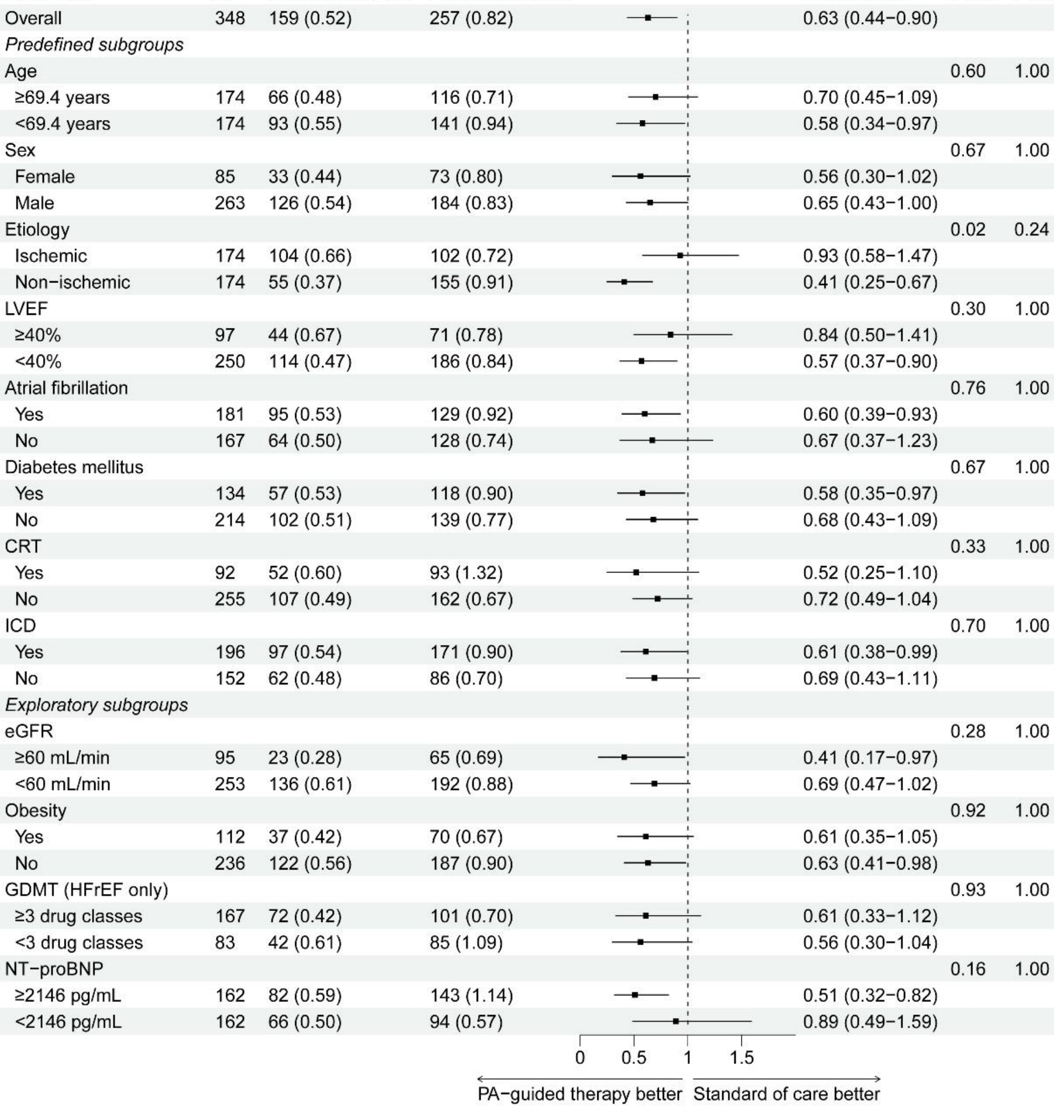

Rate: events/patient-year; CM: CardioMEMS (PA-guided therapy) group; SC: standard of care group; P int: P interaction; P int adj: Pinteraction adjusted; LVEF: left ventricular ejection fraction; CRT: cardiac resynchronization therapy; ICD: implantable cardiac defibrillator; eGFR: estimated glomerular filtration rate; GDMT: guideline-directed medical therapy; HFrEF: heart failure with a reduced ejection fraction.

Figure S2. Time to first heart failure hospitalization or cardiovascular mortality.

Subgroup	n	Events (rate) CM	Events (rate) SC	Hazard ratio	P int.	P int. adj.
Overall	348	71 (0.29)	91 (0.42)	0.71 (0.52−0.97)		
Predefined subgroups						
Age					0.20	1.00
≥69.4 years	174	34 (0.31)	47 (0.38)	0.86 (0.56−1.34)		
<69.4 years	174	37 (0.27)	44 (0.49)	0.58 (0.38−0.90)		
Sex					0.95	1.00
Female	85	17 (0.28)	26 (0.44)	0.69 (0.37−1.27)		
Male	263	54 (0.29)	65 (0.42)	0.72 (0.50−1.03)		
Etiology					0.24	1.00
Ischemic	174	41 (0.34)	43 (0.39)	0.86 (0.56−1.32)		
Non−ischemic	174	30 (0.24)	48 (0.45)	0.60 (0.38−0.94)		
LVEF					0.75	1.00
≥40%	97	20 (0.38)	29 (0.50)	0.78 (0.44−1.38)		
<40%	250	50 (0.26)	62 (0.40)	0.69 (0.48−1.00)		
Aatrial fibrillation					0.57	1.00
Yes	181	46 (0.33)	46 (0.48)	0.76 (0.50−1.14)		
No	167	25 (0.23)	45 (0.38)	0.62 (0.38−1.01)		
Diabetes mellitus					0.73	1.00
Yes	134	27 (0.31)	40 (0.46)	0.67 (0.41−1.09)		
No	214	44 (0.28)	51 (0.40)	0.75 (0.50−1.12)		
CRT					0.53	1.00
Yes	92	21 (0.31)	27 (0.56)	0.64 (0.36−1.13)		
No	255	50 (0.28)	63 (0.38)	0.76 (0.52−1.10)		
ICD					0.48	1.00
Yes	196	43 (0.32)	55 (0.42)	0.79 (0.53−1.17)		
No	152	28 (0.25)	36 (0.43)	0.62 (0.37−1.01)		
Exploratory subgroups						
eGFR					0.06	0.72
≥60 mL/min	95	9 (0.12)	23 (0.35)	0.37 (0.17−0.80)		
<60 mL/min	253	62 (0.36)	68 (0.45)	0.82 (0.58−1.16)		
Obesity					0.44	1.00
Yes	112	19 (0.24)	28 (0.42)	0.59 (0.33−1.05)		
No	236	52 (0.31)	63 (0.42)	0.77 (0.53−1.12)		
GDMT (HFrEF only)					0.96	1.00
≥3 drug classes	167	32 (0.23)	36 (0.34)	0.70 (0.43−1.12)		
<3 drug classes	83	18 (0.34)	26 (0.51)	0.73 (0.40−1.33)		
NT−proBNP					0.57	1.00
≥2146 pg/mL	162	38 (0.32)	43 (0.51)	0.66 (0.42−1.02)		
<2146 pg/mL	162	26 (0.26)	40 (0.33)	0.80 (0.49−1.31)		

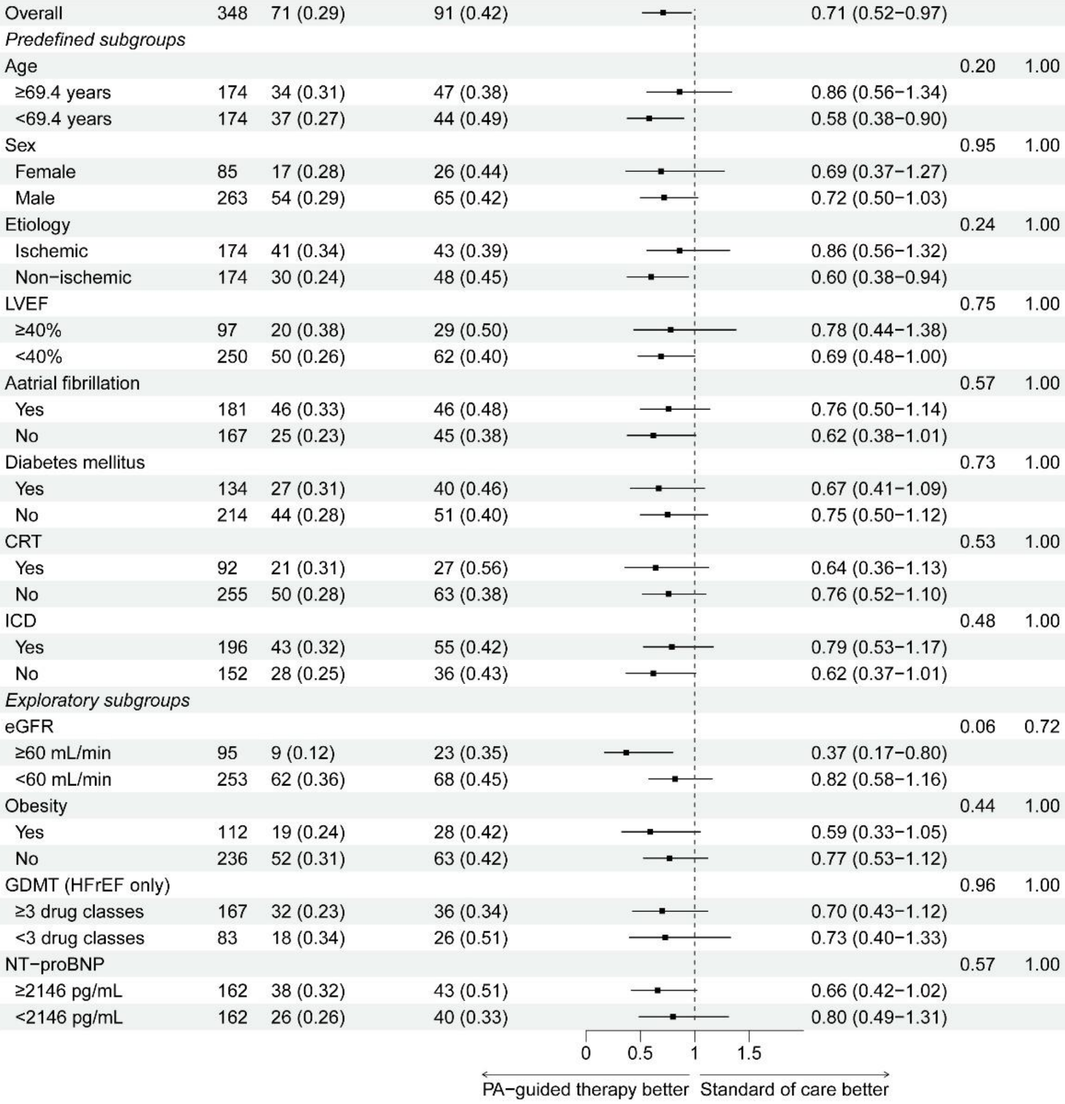

Rate: events/patient-year; CM: CardioMEMS (PA-guided therapy) group; SC: standard of care group; P int: P interaction; P int adj: Pinteraction adjusted; LVEF: left ventricular ejection fraction; CRT: cardiac resynchronization therapy; ICD: implantable cardiac defibrillator; eGFR: estimated glomerular filtration rate; GDMT: guideline-directed medical therapy; HFrEF: heart failure with a reduced ejection fraction.

Table S1. Full in- and exclusion criteria of MONITOR-HF.

Inclusion criteria	Exclusion criteria
1. Written informed consent obtained from subject aged ≥18 years.	1. Subjects with an active infection
2. Diagnosis of chronic HF* (≥3 months) in NYHA functional Class III with at least 1 HF hospitalization** or urgent visit with the necessity of intravenous (IV) diuretic therapy within 12 months of baseline visit (independent of EF %***).	2. Subjects with history of recurrent (>1) pulmonary embolism or deep vein thrombosis
3. Subjects with reduced EF (HFrEF) should be treated according to National and International (ESC) guidelines* for optimal or maximum tolerated doses of GDMT HF medication and evaluated for ICD or CRT therapy, if indicated.	3. Subjects who have had a major cardiovascular event (e.g., myocardial infarction, open heart surgery, stroke) within 2 months.
4. Subjects with a BMI ≤35 kg/m2 . Subjects with BMI >35 kg/m2 will require their chest circumference to be measured at the axillary level	4. Subjects with Cardiac Resynchronization Therapy device (CRT) implanted
5. Subjects willing and able to comply with the follow-up requirements of the study and able to comply with the daily readings.	5. Subjects with a Glomerular Filtration Rate (GFR)
	6. Subjects with complex congenital heart disease or mechanical right heart valve(s)
	7. Subjects with known pulmonary arterial hypertension (WHO category 1 or 4/5) were PA pressure are most likely not responsive to cardiac treatment.
	8. Subjects scheduled for or likely to undergo heart-transplantation or VAD within 6 months of baseline visit.
	9. Subjects with known coagulation disorders or allergy to aspirin, and/or clopidogrel

*according the ESC guidelines for heart failure 2016 definition for heart failure or subsequent versions of the ESC guidelines for heart failure 2021 during the course of the study and/or subsequent updates of recommendations in treatment of heart failure. **defined as an unscheduled HF admission longer than 6 hours and/or use of i.v. diuretics. ***patients diagnosed with heart failure according to ESC guideline definitions with either HFrEF, HFmrEF and HFpEF are eligible for participation in the study. In line with GCP, a patient cannot participate in any other intervention study (WMO-plichtig onderzoek). Additionally, a patient cannot participate in any other intervention study or other active telemonitoring programme (on HF parameters) during the study.

NYHA: New York Heart Association; HF: heart failure; EF: ejection fraction; ESC: European Society of Cardiology; ICD: implantable cardioverter-defibrillator; CRT: cardiac resynchronization therapy; BMI: body mass index; GFR: glomerular filtration rate; PA: pulmonary artery.

Table S2. Baseline characteristics of MONITOR-HF (n=348, intention-to-treat population).

	PA-guided therapy (n=176)	Standard of Care (n=172)
Age	69 (61-75)	70 (61-75)
Sex		
Male	138 (78.4%)	125 (72.7%)
Female	38 (78.4%)	47 (27.3%)
BMI, kg/m^2	27.2 (24.4-31.6)	26.8 (24.1-31.0)
Medical history		
Previous myocardial infarction	81 (46.0%)	65 (37.8%)
Previous percutaneous coronary intervention	74 (42.0%)	59 (34.3%)
Previous coronary artery bypass graft surgery	34 (19.3%)	34 (19.8%)
Diabetes	66 (37.5%)	68 (39.5%)
Cerebrovascular accident or transient ischaemic attack	29 (16.5%)	39 (22.7%)
Atrial fibrillation	100 (56.8%)	81 (47.1%)
Hypertension	102 (58.0%)	98 (57.0%)
Months since last heart failure hospitalisation	3.6 (1.2-6.4)	3.4 (1.6-6.7)
Years since heart failure diagnosis, median	3.4 (0.8-8.3)	3.8 (0.9-8.7)
Etiology		
Ischaemic	93 (52.8%)	81 (47.1%)
Heart rate, beats per min	71 (64-81)	71 (64-80)
Systolic blood pressure, mm Hg	112 (103-129)	115 (104-131)
Diastolic blood pressure, mm Hg	68 (60-75)	68 (61-76)
Left ventricular ejection fraction	30 (23-40)	30 (22-43)
<40%	127 (72.7%)	123 (71.5%)
≥40%	48 (27.3%)	49 (28.5%)
Serum creatinine (µmol/L)	127 (103-163)	124 (99-150)
eGFR, mL/min	48 (35-60)	48 (38-63)
Chronic kidney disease (eGFR <60)	131 (74.4%)	121 (70.3%)
NT-proBNP, pg/mL	2377 (837-5153)	1905 (691-4444)
Intrinsic cardiac defibrillator	94 (53.4%)	102 (59.3%)
Cardiac resynchronisation therapy	46 (26.1%)	46 (26.7%)
Medical therapy		
Beta blocker	150 (85.2%)	142 (82.6%)
Renin-angiotensin-aldosterone system inhibitor	154 (87.5%)	147 (85.5%)
Angiotensin-converting enzyme inhibitor	37 (21.0%)	32 (18.6%)
Angiotensin-receptor blocker	26 (14.8%)	26 (15.1%)
Angiotensin-receptor neprilysin inhibitor	81 (46.0%)	81 (47.1%)
Hydralazine dinitrate	10 (5.7%)	8 (4.7%)
Mineralocorticoid receptor antagonist	143 (81.3%)	144 (83.7%)
SGLT2 inhibitor	12 (6.8%)	21 (12.2%)

Loop diuretic	168 (95.5%)	167 (97.1%)
Thiazide diuretic	11 (6.3%)	10 (5.8%)
Loop and thiazide diuretic	11 (6.3%)	10 (5.8%)
Ivabradin	14 (8.0%)	10 (5.8%)
Digoxin	44 (25.0%)	39 (22.7%)

BMI: body mass index; eGFR: estimated glomerular filtration rate

Table S3. Baseline medical therapy use in HFrEF patients of MONITOR-HF (n=250, intention-to-treat population).

	PA-guided therapy (n=127)	Standard of Care (n=123)
Beta blocker	111 (87.4%)	101 (82.1%)
Renin-angiotensin-aldosterone system inhibitor	115 (90.6%)	110 (89.4%)
Angiotensin-converting enzyme inhibitor	25 (19.7%)	24 (19.5%)
Angiotensin-receptor blocker	17 (13.4%)	16 (13.0%)
Angiotensin-receptor neprilysin inhibitor	68 (53.5%)	64 (52.0%)
Hydralazine dinitrate	5 (3.9%)	6 (4.9%)
Mineralocorticoid receptor antagonist	109 (85.8%)	106 (86.2%)
SGLT2 inhibitor	11 (8.7%)	16 (13.0%)
Ivabradin	9 (9.7%)	9 (7.3%)
Digoxin	34 (26.8%)	30 (24.4%)
Loop diuretic	122 (96.1%)	119 (96.7%)
Thiazide diuretic	8 (6.3%)	8 (6.5%)
Loop and thiazide diuretic	8 (6.3%)	8 (6.5%)

Table S4. Detailed overview of quality of life endpoints.

	n	KCCQ OS at baseline (CardioMEMS HF system)	KCCQ OS at baseline (Standard Care)	KCCQ OS at 12 months (CardioMEMS HF system)	KCCQ OS at 12 months (Standard Care)	KCCQ OS mean difference at 12 months (95%CI) (between groups)	P int.	P int. adj.	KCCQ OS mean difference in change between baseline and 12 months (paired analysis) (95%CI) (CardioMEMS HF system)	KCCQ OS mean difference in change between baseline and 12 months (paired analysis) (95%CI) (Standard Care)	KCCQ OS mean difference in change between baseline and 12 months (95%CI) (between groups)	Number of missing KCCQ values at 12 months (%)	P int.	P int. adj.
Overall	279	55.8 (23.3)	54.9 (22.3)	66.1 (25.4)	56.9 (24.2)	9.19 (3.33-15.05)			7.05 (2.77-11.33)	-0.08 (-3.76-3.60)	7.13 (1.51-12.75)	69 (19.8)		
Predefined subgroups														
Age ≥69.4	131	58.1 (22.2)	53.6 (22.2)	65.1 (26.1)	57.5 (24.5)	7.55 (-1.34-16.45)	0.61	1.00	2.56 (-2.50-7.62)	2.04 (-2.80-6.89)	0.52 (-6.42-7.45)	43 (24.7)	0.03	0.33
Age <69.4	148	53.9 (24.1)	56.4 (22.6)	66.8 (25.0)	56.2 (24.1)	10.60 (2.62-18.58)			10.36 (3.93-16.78)	-2.29 (-7.93-3.36)	12.64 (4.16-21.13)	26 (14.9)		
Female	74	53.2 (22.9)	49.3 (22.5)	56.8 (28.0)	50.9 (25.9)	5.90 (-6.81-18.6)	0.56	1.00	2.31 (-6.18-10.79)	0.55 (-6.86-7.96)	1.76 (-9.31-12.83)	11 (12.9)	0.27	1.00
Male	205	56.6 (23.4)	57.0 (22.0)	69.0 (23.9)	59.2 (23.3)	9.77 (3.27-16.26)			8.57 (3.57-13.56)	-0.33 (-4.62-3.97)	8.89 (2.34-15.44)	58 (22.1)		
Ischemic etiology	131	57.1 (24.5)	52.6 (22.1)	67.9 (25.6)	58.2 (19.9)	9.63 (1.70-14.56)	0.86	1.00	6.18 (-0.22-12.57)	3.19 (-1.81-8.19)	2.99 (-5.06-11.03)	43 (24.7)	0.18	1.00

Non-ischemic etiology	148	54.5 (21.8)	56.9 (22.5)	64.3 (25.2)	55.8 (27.3)	8.55 (0.00-17.11)			7.89 (2.03-13.76)	-2.74 (-8.06-2.58)	10.63 (2.78-18.49)	26 (14.9)		
LVEF ≥40	79	56.3 (22.8)	52.6 (20.7)	62.9 (27.9)	51.0 (22.9)	11.9 (0.17-23.65)	0.52	1.00	3.12 (-4.99-11.23)	-2.09 (-7.34-3.17)	5.20 (-4.32-14.73)	18 (18.6)	0.71	1.00
LVEF <40	200	55.8 (23.6)	55.8 (23.0)	67.2 (24.5)	59.5 (24.5)	7.69 (0.86-14.52)			8.41 (3.33-13.5)	0.81 (-4.01-5.63)	7.60 (0.64-14.56)	50 (20.0)		
AF	141	57.7 (24.1)	54.3 (23.0)	64.3 (26.4)	55.7 (24.8)	8.57 (0.05-17.08)	0.77	1.00	2.84 (-2.70-8.38)	0 (-6.06-6.05)	2.84 (-5.30-10.98)	40 (22.1)	0.10	0.80
No AF	138	53.4 (22.1)	55.4 (21.9)	68.2 (24.2)	57.9 (23.8)	10.3 (2.13-18.47)			12.10 (5.49-18.71)	-0.14 (-4.68-4.40)	12.24 (4.29-20.20)	29 (17.4)		
DM	105	56.0 (25.2)	50.7 (21.7	63.6 (25.5)	57.0 (25.9)	6.61 (-3.41-16.63)	0.52	1.00	3.09 (-4.92-11.11)	6.16 (0.49-11.83)	-3.06 (-12.77-6.65)	29 (21.6)	0.005	0.06
No DM	174	55.7 (22.2)	57.6 (22.5)	67.4 (25.4)	56.8 (23.2)	10.58 (3.30-17.86)			9.16 (4.12-14.21)	-4.26 (-8.98-0.47)	13.42 (6.56-20.28)	40 (18.7)		
CRT	72	57.0 (21.9)	55.3 (20.4)	60.8 (25.2)	56.1 (25.0)	4.69 (-7.10-16.48)	0.38	1.00	0.73 (-7.20-8.67)	-1.68 (-9.39-6.03)	2.41 (-8.45-13.28)	20 (21.7)	0.32	1.00
No CRT	206	55.4 (23.8)	54.9 (23.1)	68.0 (25.3)	57.4 (24.0)	10.61 (3.81-17.41)			9.42 (4.34-14.50)	0.56 (-3.73-4.84)	8.86 (2.26-15.47)	49 (19.2)		
ICD	160	56.9 (22.6)	57.1 (21.4)	65.7 (25.2)	58.6 (24.1)	7.10 (-0.65-14.84)	0.40	1.00	7.75 (1.83-13.66)	-1.49 (-6.26-3.27)	9.24 (1.70-16.78)	36 (18.4%)	0.38	1.00
No ICD	119	54.6 (24.1)	51.7 (23.4)	66.5 (25.8)	54.4 (24.4)	12.15 (3.03-21.27)			6.18 (-0.18-12.55)	1.97 (-3.95-7.90)	4.21 (-4.39-12.81)	33 (21.7)		
Exploratory subgroups														
eGFR ≥60	81	56.9 (22.9)	53.1 (24.9)	64.2 (24.2)	59.9 (23.7)	4.25 (-6.42-14.92)	0.29	1.00	8.17 (1.23-15.12)	3.66 (-2.75-10.07)	4.51 (-4.79-13.82)	14 (14.7)	0.55	1.00

eGFR <60	198	55.5 (23.5)	55.7 (21.2)	66.8 (25.9)	55.6 (24.4)	11.23 (4.16-18.3)			6.61 (1.25-11.98)	-1.67 (-6.19-2.85)	8.28 (1.31-15.26)	55 (21.7)		
Obesity	91	51.5 (21.6)	53.2 (22.6)	64.4 (25.8)	53.4 (24.6)	10.98 (0.40-21.57)	0.66	1.00	7.76 (0.64-14.89)	-0.05 (-7.10-7.00)	7.81 (-2.07-17.70)	21 (18.8)	0.87	1.00
No obesity	188	58.0 (23.9)	55.6 (22.3)	66.8 (25.3)	58.6 (24.0)	8.16 (1.06-15.26)			6.73 (1.33-12.13)	-0.09 (-4.42-4.24)	6.82 (-0.06-13.70)	48 (20.3)		
GDMT ≥3	144	57.5 (21.4)	58.6 (21.8)	67.9 (23.7)	59.0 (24.9)	8.91 (0.90-16.93)	0.55	1.00	8.11 (2.51-13.71)	-1.77 (-7.46-3.91)	9.88 (1.97-17.80)	23 (13.8)	0.40	1.00
GDMT <3	56	51.7 (28.2)	51.1 (24.4)	64.7 (27.4)	60.4 (23.9)	4.24 (-10.02-18.50)			9.41 (-3.13-21.94)	6.22 (-2.92-15.35)	3.19 (-11.98-18.36)	27 (32.5)		
NTproBNP ≥2146	119	53.6 (23.0)	53.3 (21.5)	64.1 (25.7)	55.5 (23.3)	8.60 (-0.32-17.51)	0.97	1.00	7.38 (0.09-14.66)	-0.31 (-6.70-6.09)	7.69 (-1.91-17.28)	43 (26.5)	0.85	1.00
NTproBNP <2146	138	57.7 (22.6)	57.5 (22.5)	67.0 (25.4)	58.6 (25.4)	8.38 (-0.26-17.01)			6.02 (0.80-11.24)	-0.57 (-5.11-3.98)	6.59 (-0.27-13.44)	24 (14.8)		

KCCQ OS: Kansas City Cardiomyopathy Questionnaire overall summary score; P int: P interaction; P int adj: Pinteraction adjusted; LVEF: left ventricular ejection fraction; AF: atrial fibrillation; DM: diabetes mellitus; CRT: cardiac resynchronization therapy; ICD: implantable cardiac defibrillator; eGFR: estimated glomerular filtration rate; GDMT: guideline-directed medical therapy; HFrEF: heart failure with a reduced ejection fraction.

Table S5. Detailed overview of clinical endpoints.

	n	Total HFH (rate/pty) (CardioMEMS HF system)	Total HFH (rate/pty) (Standard Care)	Total HFH HR (95%CI)	P int.	P int. adj.	Total HFH and all-cause mortality (rate/pty) (CardioMEMS HF system)	Total HFH and all-cause mortality (rate/pty) (Standard Care)	Total HFH and all-cause mortality HR (95%CI)	P int.	P int. adj.
Overall	348	117 (0.38)	212 (0.68)	0.56 (0.38-0.84)			159 (0.52)	257 (0.82)	0.63 (0.44-0.90)		
Predefined subgroups											
Age ≥69.4	174	45 (0.33)	85 (0.52)	0.64 (0.38-1.08)	0.50	1.00	66 (0.48)	116 (0.71)	0.70 (0.45-1.09)	0.60	1.00
Age <69.4	174	72 (0.42)	127 (0.85)	0.49 (0.28-0.87)			93 (0.55)	141 (0.94)	0.58 (0.34-0.97)		
Female	85	27 (0.36)	65 (0.72)	0.51 (0.28-0.94)	0.74	1.00	33 (0.44)	73 (0.80)	0.56 (0.30-1.02)	0.67	1.00
Male	263	90 (0.39)	147 (0.66)	0.58 (0.36-0.96)			126 (0.54)	184 (0.83)	0.65 (0.43-1.00)		
Ischemic etiology	174	75 (0.48)	76 (0.54)	0.89 (0.52-1.55)	0.02	0.24	104 (0.66)	102 (0.72)	0.93 (0.58-1.47)	0.02	0.24
Non-ischemic etiology	174	42 (0.28)	136 (0.80)	0.36 (0.22-0.59)			55 (0.37)	155 (0.91)	0.41 (0.25-0.67)		
LVEF ≥40	97	33 (0.50)	63 (0.69)	0.70 (0.40-1.23)	0.42	1.00	44 (0.67)	71 (0.78)	0.84 (0.50-1.41)	0.30	1.00
LVEF <40	250	84 (0.35)	149 (0.68)	0.53 (0.31-0.88)			114 (0.47)	186 (0.84)	0.57 (0.37-0.90)		
AF	181	72 (0.40)	108 (0.77)	0.54 (0.34-0.88)	0.89	1.00	95 (0.53)	129 (0.92)	0.60 (0.39-0.93)	0.76	1.00
No AF	167	45 (0.35)	104 (0.60)	0.57 (0.28-1.18)			64 (0.50)	128 (0.74)	0.67 (0.37-1.23)		
DM	134	34 (0.32)	96 (0.73)	0.42 (0.23-0.77)	0.27	1.00	57 (0.53)	118 (0.90)	0.58 (0.35-0.97)	0.67	1.00
No DM	214	83 (0.42)	116 (0.64)	0.67 (0.40-1.12)			102 (0.51)	139 (0.77)	0.68 (0.43-1.09)		

CRT	92	42 (0.48)	74 (1.05)	0.54 (0.23-1.24)	0.66	1.00	52 (0.60)	93 (1.32)	0.52 (0.25-1.10)	0.33	1.00
No CRT	255	75 (0.34)	136 (0.56)	0.60 (0.39-0.91)			107 (0.49)	162 (0.67)	0.72 (0.49-1.04)		
ICD	196	76 (0.43)	143 (0.76)	0.57 (0.33-0.98)	1.00	1.00	97 (0.54)	171 (0.90)	0.61 (0.38-0.99)	0.70	1.00
No ICD	152	41 (0.32)	69 (0.56)	0.57 (0.33-0.97)			62 (0.48)	86 (0.70)	0.69 (0.43-1.11)		
Exploratory subgroups											
eGFR ≥60	95	19 (0.23)	55 (0.58)	0.40 (0.16-0.99)	0.43	1.00	23 (0.28)	65 (0.69)	0.41 (0.17-0.97)	0.28	1.00
eGFR <60	253	98 (0.44)	157 (0.72)	0.61 (0.39-0.95)			136 (0.61)	192 (0.88)	0.69 (0.47-1.02)		
Obesity	112	23 (0.26)	63 (0.60)	0.41 (0.22-0.77)	0.34	1.00	37 (0.42)	70 (0.67)	0.61 (0.35-1.05)	0.92	1.00
No obesity	236	94 (0.43)	149 (0.72)	0.61 (0.38-1.01)			122 (0.56)	187 (0.90)	0.63 (0.41-0.98)		
GDMT ≥3	167	54 (0.32)	87 (0.60)	0.53 (0.26-1.06)	0.92	1.00	72 (0.42)	101 (0.70)	0.61 (0.33-1.12)	0.93	1.00
GDMT <3	83	30 (0.44)	64 (0.82)	0.53 (0.27-1.06			42 (0.61)	85 (1.09)	0.56 (0.30-1.04)		
NTproBNP ≥2146	162	54 (0.39)	116 (0.93)	0.42 (0.25-0.70)	0.12	1.00	82 (0.59)	143 (1.14)	0.51 (0.32-0.82)	0.16	1.00
NTproBNP <2146	162	52 (0.40)	80 (0.48)	0.82 (0.42-1.58)			66 (0.50)	94 (0.57)	0.89 (0.49-1.59)		

HFH: heart failure hospitalization; pty: patient-year; HR: hazard ratio;P int: P interaction; P int adj: P interaction adjusted; LVEF: left ventricular ejection fraction; AF: atrial fibrillation; DM: diabetes mellitus; CRT: cardiac resynchronization therapy; ICD: implantable cardiac defibrillator; eGFR: estimated glomerular filtration rate; GDMT: guideline-directed medical therapy; HFrEF: heart failure with a reduced ejection fraction.

Table S5. Detailed overview of clinical endpoints (continued).

	n	Time to first HFH (rate) CardioMEMS HF system	Time to first HFH (rate) Standard Care	Time to first HFH HR (95%CI)	P int.	P int. adj.	Time to first HFH or CV mortality (rate) (CardioMEMS HF system)	Time to first HFH or CV mortality (rate) (Standard Care)	Time to first HFH or CV mortality HR (95%CI)	P int.	P int. adj.
Overall	348	63 (0.25)	85 (0.40)	0.67 (0.49-0.93)			71 (0.29)	91 (0.42)	0.71 (0.52-0.97)		
Predefined subgroups											
Age ≥69.4	174	15 (0.14)	22 (0.18)	0.82 (0.51-1.32)	0.22	1.00	34 (0.31)	47 (0.38)	0.86 (0.56-1.34)	0.20	1.00
Age <69.4	174	25 (0.18)	25 (0.28)	0.55 (0.35-0.86)			37 (0.27)	44 (0.49)	0.58 (0.38-0.90)		
Female	85	16 (0.26)	26 (0.44)	0.64 (0.35-1.21)	0.91	1.00	17 (0.28)	26 (0.44)	0.69 (0.37-1.27)	0.95	1.00
Male	263	47 (0.25)	59 (0.38)	0.69 (0.47-1.01)			54 (0.29)	65 (0.42)	0.72 (0.50-1.03)		
Ischemic etiology	174	34 (0.28)	37 (0.34)	0.83 (0.52-1.32)	0.27	1.00	41 (0.34)	43 (0.39)	0.86 (0.56-1.32)	0.24	1.00
Non-ischemic etiology	174	29 (0.23	48 (0.45)	0.58 (0.36-0.91)			30 (0.24)	48 (0.45)	0.60 (0.38-0.94)		
LVEF ≥40	97	19 (0.36)	29 (0.50)	0.74 (0.41-1.32)	0.81	1.00	20 (0.38)	29 (0.50)	0.78 (0.44-1.38)	0.75	1.00
LVEF <40	250	44 (0.23)	56 (0.36)	0.67 (0.45-1.00)			50 (0.26)	62 (0.40)	0.69 (0.48-1.00)		
AF	181	41 (0.30)	45 (0.47)	0.69 (0.45-1.05)	0.78	1.00	46 (0.33)	46 (0.48)	0.76 (0.50-1.14)	0.57	1.00
No AF	167	22 (0.20)	40 (0.34)	0.61 (0.36-1.03)			25 (0.23)	45 (0.38)	0.62 (0.38-1.01)		
DM	134	23 (0.26)	38 (0.44)	0.60 (0.36-1.01)	0.54	1.00	27 (0.31)	40 (0.46)	0.67 (0.41-1.09)	0.73	1.00
No DM	214	40 (0.25)	47 (0.37)	0.74 (0.48-1.13)			44 (0.28)	51 (0.40)	0.75 (0.50-1.12)		

CRT	92	19 (0.28)	25 (0.52)	0.62 (0.34-1.14)	0.62	1.00	21 (0.31)	27 (0.56)	0.64 (0.36-1.13)	0.53	1.00
No CRT	255	44 (0.24)	59 (0.35)	0.71 (0.48-1.05)			50 (0.28)	63 (0.38)	0.76 (0.52-1.10)		
ICD	196	39 (0.29)	52 (0.39)	0.75 (0.50-1.14)	0.45	1.00	43 (0.32)	55 (0.42)	0.79 (0.53-1.17)	0.48	1.00
No ICD	152	24 (0.21)	33 (0.40)	0.57 (0.34-0.97)			28 (0.25)	36 (0.43)	0.62 (0.37-1.01)		
Exploratory subgroups											
eGFR ≥60	95	8 (0.11)	22 (0.34	0.34 (0.15-0.77)	0.07	0.84	9 (0.12)	23 (0.35)	0.37 (0.17-0.80)	0.06	0.72
eGFR <60	253	55 (0.32)	63 (0.42)	0.79 (0.55-1.13)			62 (0.36)	68 (0.45)	0.82 (0.58-1.16)		
Obesity	112	15 (0.19)	28 (0.42)	0.46 (0.25-0.87)	0.16	1.00	19 (0.24)	28 (0.42)	0.59 (0.33-1.05)	0.44	1.00
No obesity	236	48 (0.28)	57 (0.38)	0.79 (0.53-1.15)			52 (0.31)	63 (0.42)	0.77 (0.53-1.12)		
GDMT ≥3	167	27 (0.19)	32 (0.30)	0.66 (0.39-1.10)	0.82	1.00	32 (0.23)	36 (0.34)	0.70 (0.43-1.12)	0.96	1.00
GDMT <3	83	17 (0.32)	24 (0.48)	0.74 (0.40-1.39)			18 (0.34)	26 (0.51)	0.73 (0.40-1.33)		
NTproBNP ≥2146	162	32 (0.27)	39 (0.46)	0.61 (0.38-0.97)	0.49	1.00	38 (0.32)	43 (0.51)	0.66 (0.42-1.02)	0.57	1.00
NTproBNP <2146	162	24 (0.24)	38 (0.31)	0.77 (0.46-1.29)			26 (0.26)	40 (0.33)	0.80 (0.49-1.31)		

HFH: heart failure hospitalization; CV: cardiovascular; pty: patient-year; HR: hazard ratio;P int: P interaction; P int adj: P interaction adjusted; LVEF: left ventricular ejection fraction; AF: atrial fibrillation; DM: diabetes mellitus; CRT: cardiac resynchronization therapy; ICD: implantable cardiac defibrillator; eGFR: estimated glomerular filtration rate; GDMT: guideline-directed medical therapy; HFrEF: heart failure with a reduced ejection fraction.

Table S6. Detailed overview of pulmonary artery pressure endpoints.

	n	Mean PAP AUC at 12 months	P int.	P int. adj.	mPAP at baseline	mPAP at 12 months	mPAP mean change (paired analysis) (95%CI)	P int.	P int. adj.
Overall	168	-1624 (2003)			33.3 (10.6)	24.9 (9.4)	-6.46 (-7.97- -4.94)		
Predefined subgroups									
Age ≥69.4	74	-1728 (1971)	0.55	1.00	35.4 (9.89)	27.6 (9.89)	-6.59 (-9.10- -4.08)	0.89	1.00
Age <69.4	94	-1543 (2035)			31.6 (10.9)	23.0 (8.51)	-6.36 (-8.29- -4.43)		
Female	37	-1094 (1540)	0.03	0.33	30.8 (11.6)	25.1 (10.3)	-3.86 (-5.79- -1.93)	0.01	0.12
Male	131	-1775 (2097)			34.0 (10.3)	24.9 (9.12)	-7.25 (-9.13 - -5.38)		
Ischemic etiology	88	-1636 (2074)	0.93	1.00	35.0 (10.9)	25.8 (8.96)	-6.44 (-8.73- -4.15)	0.98	1.00
Non-ischemic etiology	80	-1610 (1934)			31.4 (10.0)	24.0 (9.77)	-6.48 (-8.52- -4.44)		
LVEF ≥40	46	-1104 (1703)	0.02	0.24	32.6 (9.82)	25.7 (8.69)	-4.32 (-7.07 - -1.57)	0.08	0.80
LVEF <40	121	-1830 (2079)			33.4 (10.9)	24.7 (9.62)	-7.18 (-8.98- -5.37)		
AF	95	-1576 (1908)	0.73	1.00	34.6 (9.85)	26.7 (9.59)	-6.11 (-8.11- -4.12)	0.62	1.00
No AF	73	-1686 (2131)			31.6 (11.4)	22.6 (8.63)	-6.9 (-9.30- -4.50)		
DM	61	-1778 (1959)	0.45	1.00	34.5 (9.42)	27.0 (8.95)	-5.63 (-8.07- -3.19)	0.42	1.00
No DM	107	-1535 (2032)			32.6 (11.2)	23.8 (9.46)	-6.90 (-8.86- -4.94)		
CRT	45	-1493 (1956)	0.60	1.00	33.0 (9.34)	25.8 (8.47)	-5.89 (-8.73- -3.05)	0.65	1.00
No CRT	123	-1672 (2027)			33.4 (11.1)	24.6 (9.69)	-6.66 (-8.48- -4.84)		

ICD	92	-1722 (1985)	0.49	1.00	33.6 (10.2)	24.4 (8.59)	-7.31 (-9.49- -5.14)	0.23	1.00
No ICD	76	-1503 (2033)			32.9 (11.2)	25.5 (10.2)	-5.46 (-7.58- -3.33)		
Exploratory subgroups									
Baseline mPAP >25	93	-2112 (2295)	0.0002	0.003	39.6 (7.88)	29.3 (8.72)	-9.18 (-11.72- -6.64)	0.0004	0.006
Baseline mPAP ≤25	69	-987 (1376)			25.0 (8.22)	20.8 (8.38)	-3.56 (-5.24- -1.87)		
eGFR ≥60	43	-1374 (1541)	0.27	1.00	27.8 (10.5)	20.6 (8.26)	-5.04 (-6.90- -3.18)	0.15	1.00
eGFR <60	125	-1711 (2139)			35.2 (10.0)	26.7 (9.26)	-7.03 (-9.02- -5.03)		
Obesity	54	-1428 (1756)	0.36	1.00	32.8 (9.94)	27.1 (7.27)	-4.08 (-6.22- -1.93)	0.02	0.22
No obesity	114	-1715 (2110)			33.5 (10.9)	24.0 (10.0)	-7.46 (-9.39- -5.52)		
GDMT ≥3	88	1734 (2014)	0.43	1.00	32.5 (10.8)	24.3 (8.73)	-6.52 (-8.52- -4.51)	0.26	1.00
GDMT <3	33	2088 (2257)			35.8 (11.0)	25.7 (12.0)	-9.09 (-13.27- -4.93)		
NTproBNP ≥2146	82	-2183 (2166)	0.001	0.01	37.2 (9.41)	26.3 (8.54)	-9.15 (-11.45- -6.84)	0.003	0.04
NTproBNP <2146	73	-1147 (1767)			29.5 (11.0)	23.1 (10.2)	-4.38 (-6.55- -2.22)		
PVR >2	65	-2038 (2087)	0.09	0.90	37.5 (9.25)	27.6 (8.46)	-8.49 (-10.94- -6.05)	0.11	0.99
PVR ≤2	88	-1462 (2000)			30.5 (10.2)	23.2 (9.67)	-5.85 (-8.03- -3.68)		

PAP AUC: pulmonary artery pressure area under the curve (mmHg.days); mPAP: mean pulmonary artery pressure (mmHg); 12M: 12 months; P int: P interaction; P int adj: P interaction adjusted; LVEF: left ventricular ejection fraction; AF: atrial fibrillation; DM: diabetes mellitus; CRT: cardiac resynchronization therapy; ICD: implantable cardiac defibrillator; eGFR: estimated glomerular filtration rate; GDMT: guideline-directed medical therapy; HFrEF: heart failure with a reduced ejection fraction; PVR: peripheral vascular resistance.
